# Supplementary material for: Yuanhuacine Is a Potent and Selective Inhibitor of the Basal-Like 2 Subtype of Triple Negative Breast Cancer with Immunogenic Potential
Source: Cancers (Basel). 2021 Jun 7;13(11):2834. doi: 10.3390/cancers13112834 (PMC8201195; doi:10.3390/cancers13112834)
Supplement: Supplementary file 1 [file cancers-13-02834-s001.zip › cancers-1237536-supplementary.pdf]

cancers

MDPI

Article

Supplementary Materials: Yuanhuacine Is a Potent and Selective Inhibitor of the Basal-Like 2 Subtype of Triple Negative Breast Cancer with Immunogenic Potential

Charles S. Fermaintt, Thilini Peramuna, Shengxin Cai, Leila Takahashi-Ruiz, Jacob Nathaniel Essif, Corena V. Grant, Barry R. O'Keefe, Susan L. Mooberry, Robert H. Cichewicz and April L. Risinger

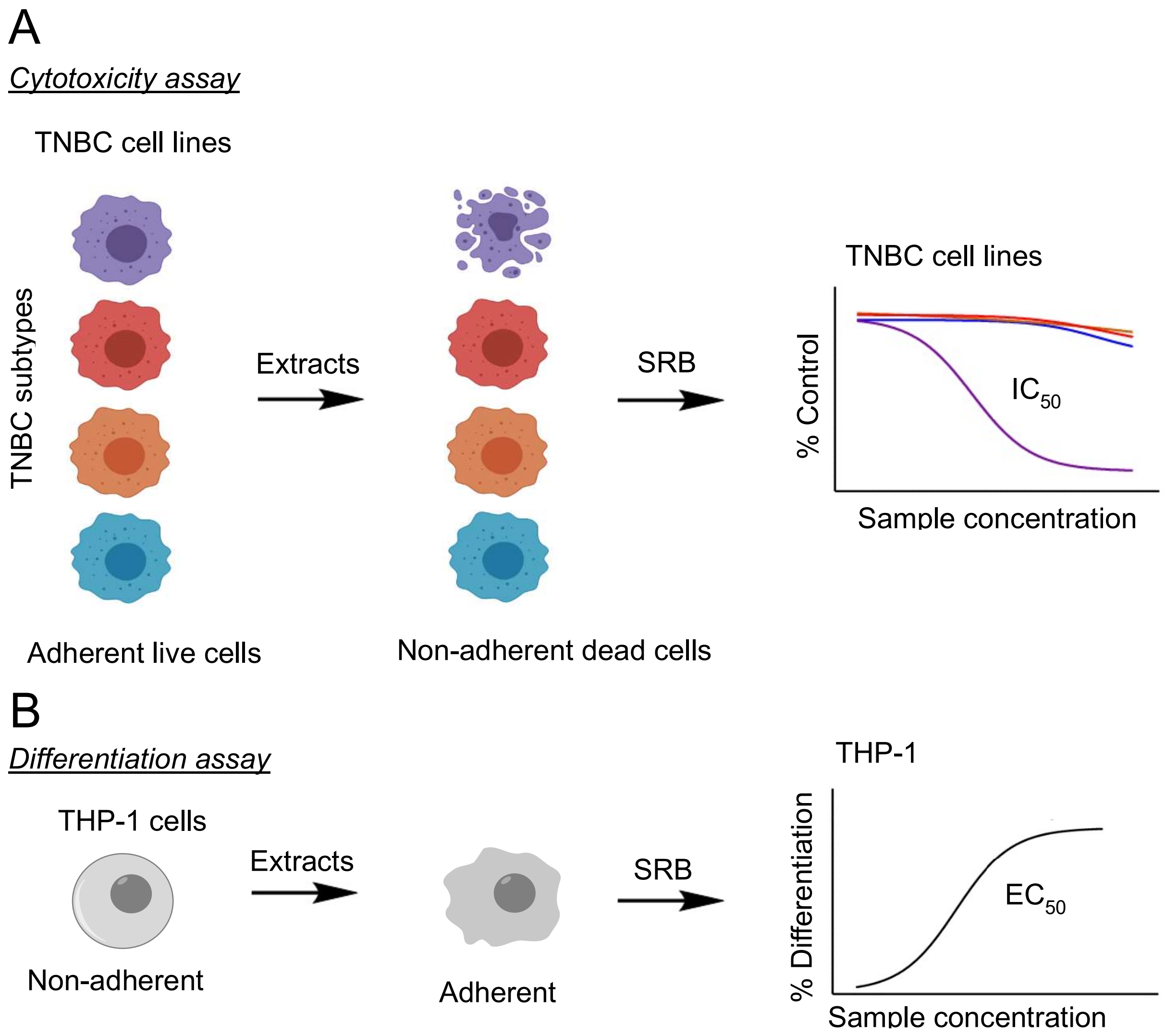

Figure S1. TNBC selectivity and THP-1 differentiation screening assays. (**A**) Schematic diagram illustrating the sulforhodamine B (SRB) based cytotoxicity screening assay against cell lines representing molecularly distinct TNBC subtypes. (**B**) Schematic diagram illustrating the sulforhodamine B (SRB) based THP-1 differentiation screening assay where non-adherent THP-1 cells in the monocytic state become adherent myeloid cells (e.g. macrophages and dendritic cells) in response to immunogenic ligands.

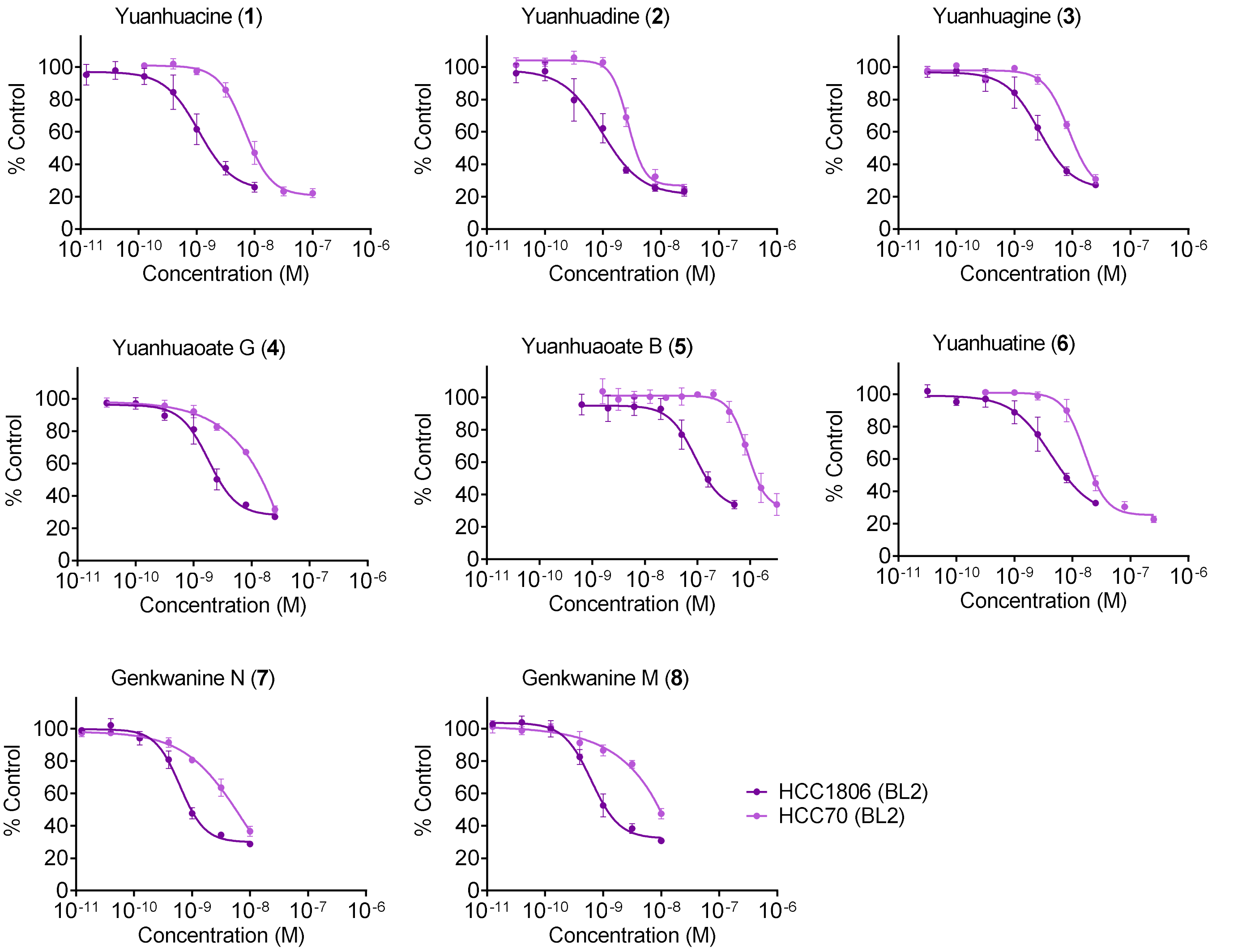

Figure S2. Concentration response curves for the growth of HCC1806 and HCC70 cells treated for 48 h with compounds 1-8.

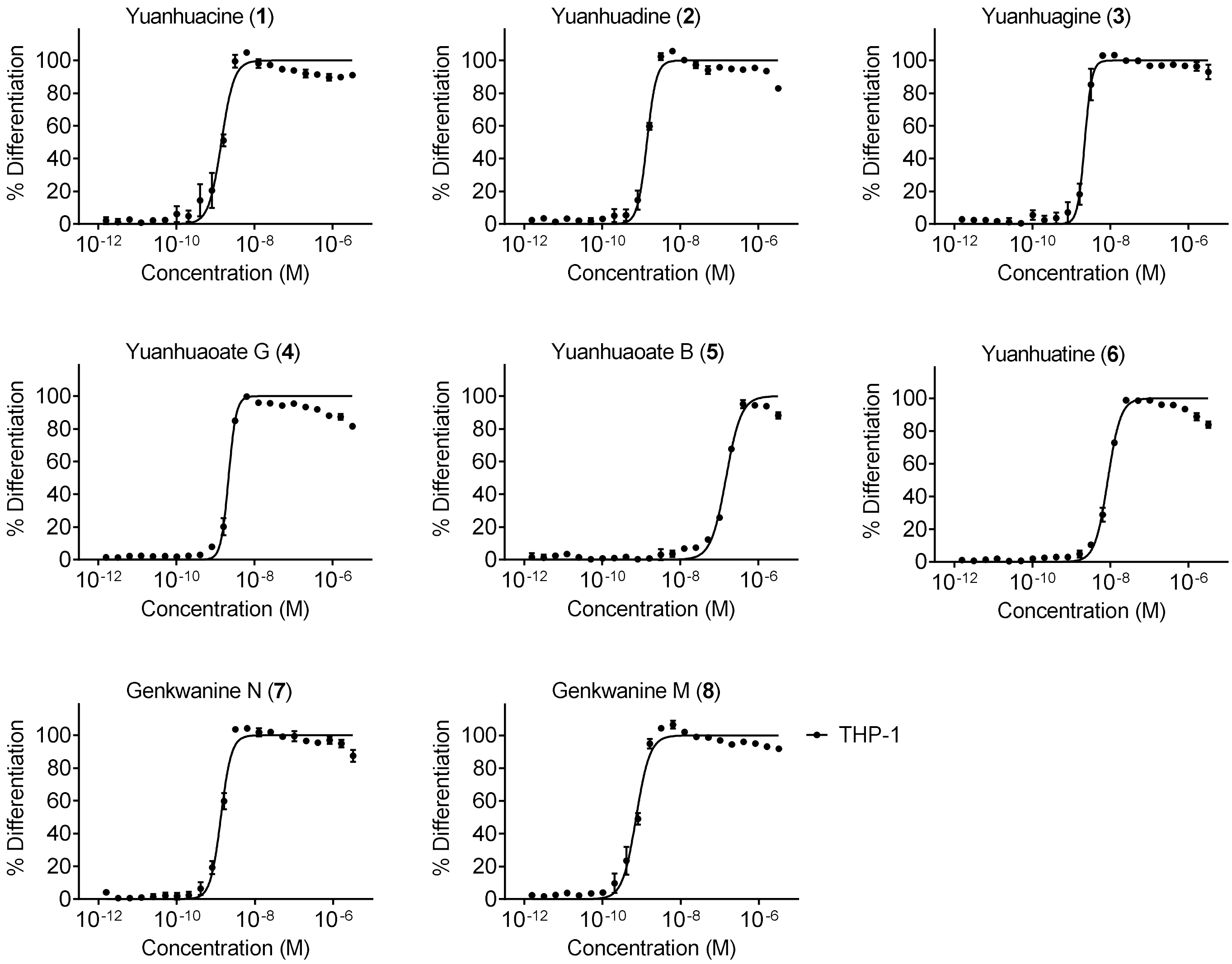

Figure S3. Concentration response curves for the differentiation of THP-1 cell treated for 24 h with compounds 1-8.

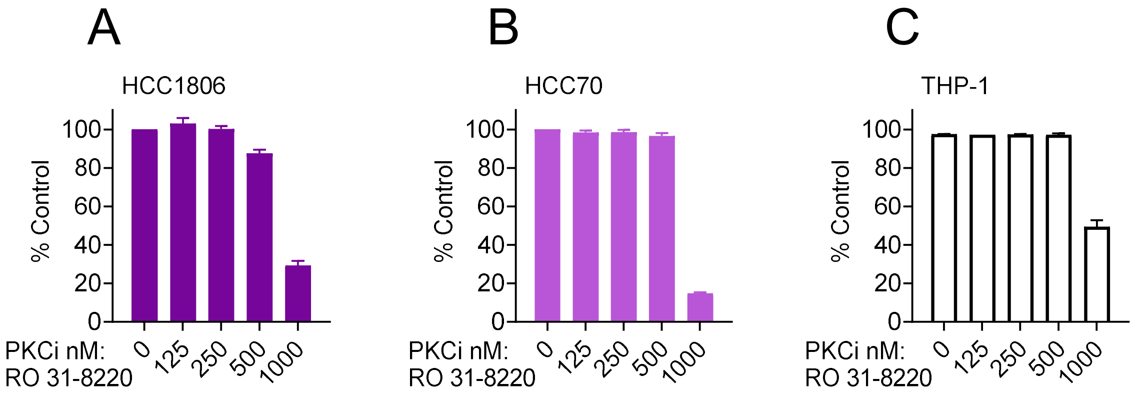

Figure S4. Growth of cells treated with the PKC inhibitor Ro 31-8220. Growth of (**A**) HCC1806 or (**B**) HCC70 cells when treated with the PKC inhibitor Ro 31-8220 (PKCi) for 48 h as determined by the SRB assay. (**C**) Caspase 3/7 cleavage in THP-1 cells treated with the PKC inhibitor Ro 31-8220 for 24 h.

Table S1. List of DNA oligonucleotides used in this study. All oligonucleotides were purchased from Sigma-Aldrich and validated by performing a Primer-Blast (https://www.ncbi.nlm.nih.gov/tools/primer-blast/index.cgi, accessed on 5 March 2021).

Gene Name	Forward and reverse oligonucleotide sequence (5′ → 3′) used in qRT-PCR analyses
Human GAPDH	GCAAATTCCATGGCACCGT
	TCGCCCCACTTGATTTTGG
Human IFNγ	CTAATTATTCGGTAACTGACTTGA
	ACAGTTCAGCCATCACTTGGA
Human IL-12	CATGGTGGATGCCGTTCACA
	ACCTCCACCTGCCGAGAATT
Human IL-10	GCTGGAGGACTTTAAGGGTTACCT
	CTTGATGTCTGGGTCTTGGTTCTC
Mouse GAPDH	TTCACCACCATGGAGAAGGC
	GGCATGGACTGTGGTCATGA
Mouse IL-12	AGAAAGGTGCGTTCCTCGTAG
	AGCCAACCAAGCAGAAGACAG
